# Focal T2 and FLAIR hyperintensities within the infarcted area: A suitable marker for patient selection for treatment?

**DOI:** 10.1371/journal.pone.0185158

**Published:** 2017-09-28

**Authors:** Julia Meisterernst, Pascal P. Klinger-Gratz, Lars Leidolt, Matthias F. Lang, Gerhard Schroth, Pasquale Mordasini, Mirjam R. Heldner, Marie-Luise Mono, Rebekka Kurmann, Monika Buehlmann, Urs Fischer, Marcel Arnold, Jan Gralla, Heinrich P. Mattle, Marwan El-Koussy, Simon Jung

**Affiliations:** 1 Department of Neurology, Inselspital, University Hospital Bern and University of Bern, Bern, Switzerland; 2 Department of Diagnostic and Interventional Neuroradiology, Inselspital, University Hospital Bern and University of Bern, Bern, Switzerland; 3 Department of Radiology, University Hospital of Basel, University of Basel, Basel, Switzerland; 4 Department of Diagnostic, Interventional and Pediatric Radiology, Inselspital, Bern University Hospital, University of Bern, Bern, Switzerland; University of Münster, GERMANY

## Abstract

**Background and purpose:**

Some authors use FLAIR imaging to select patients for stroke treatment. However, the effect of hyperintensity on FLAIR images on outcome and bleeding has been addressed in only few studies with conflicting results.

**Methods:**

466 patients with anterior circulation strokes were included in this study. They all were examined with MRI before intravenous or endovascular treatment. Baseline data and 3 months outcome were recorded prospectively. Focal T2 and FLAIR hyperintensities within the ischemic lesion were evaluated by two raters, and the PROACT II classification was applied to assess bleeding complications on follow up imaging. Logistic regression analysis was used to determine predictors of bleeding complications and outcome and to analyze the influence of T2 or FLAIR hyperintensity on outcome.

**Results:**

Focal hyperintensities were found in 142 of 307 (46.3%) patients with T2 weighted imaging and in 89 of 159 (56%) patients with FLAIR imaging. Hyperintensity in the basal ganglia, especially in the lentiform nucleus, on T2 weighted imaging was the only independent predictor of any bleeding after reperfusion treatment (33.8% in patients with vs. 18.2% in those without; p = 0.003) and there was a non-significant trend for more bleedings in patients with FLAIR hyperintensity within the basal ganglia (p = 0.069). However, there was no association of hyperintensity on T2 weighted or FLAIR images and symptomatic bleeding or worse outcome.

**Conclusion:**

Our results question the assumption that T2 or FLAIR hyperintensities within the ischemic lesion should be used to exclude patients from reperfusion therapy, especially not from endovascular treatment.

## Introduction

Focal hyperintensities on T2 weighted spin echo or fluid-attenuated inversion recovery (FLAIR) imaging in the region of diffusion restriction on diffusion weighted imaging (DWI) have been identified as a tissue marker of the ischemic lesion age. Such hyperintensities are regarded as a new tool to select stroke patients with unknown symptom onset for treatment with intravenous thrombolysis (IVT).[1–6] Reperfusion therapy based on mismatch between DWI and FLAIR images, i.e. no FLAIR hyperintensity within the DWI lesion, has been shown to be feasible and safe when symptom onset of stroke is unknown.[7, 8]

The exclusion of patients with FLAIR hyperintensity from reperfusion treatment assumes an adverse outcome of such patients. To date, however, only few studies addressed this issue. Four studies showed a higher rate of haemorrhagic transformation (HT) or symptomatic intracerebral haemorrhage (sICH) in patients with FLAIR hyperintensity with or without IVT[9–12], whereas one study did not.[13] Results on outcome are even more conflicting. Two studies found a negative association of FLAIR hyperintensity on clinical outcome after IVT[2, 14], but two other studies using IVT[15] and endovascular therapy (EVT)[16] did not. Therefore, the question arises whether T2 or FLAIR hyperintensities represent a suitable marker to exclude patients from reperfusion therapy in any time window.

The aim of this study was to analyse the effect of focal T2 weighted and FLAIR hyperintensity on baseline imaging and the impact of the infarct localization on bleeding and clinical outcome in a large cohort of patients treated by IVT, EVT or both.

## Patients and methods

### Patients and treatment

The present study included patients of the Bernese stroke registry, a prospectively collected database. Some of its aspects have been reported previously.[17–22] Patients were included in this analysis if: 1) diagnosis of ischemic stroke was established with MRI between 2004 and 2014, 2) the infarct was located in the anterior circulation, 3) IVT, EVT or bridging IVT and EVT was performed, and 4) a control CT or MRI scan was carried out at 24h after treatment.

**Fig 1:**
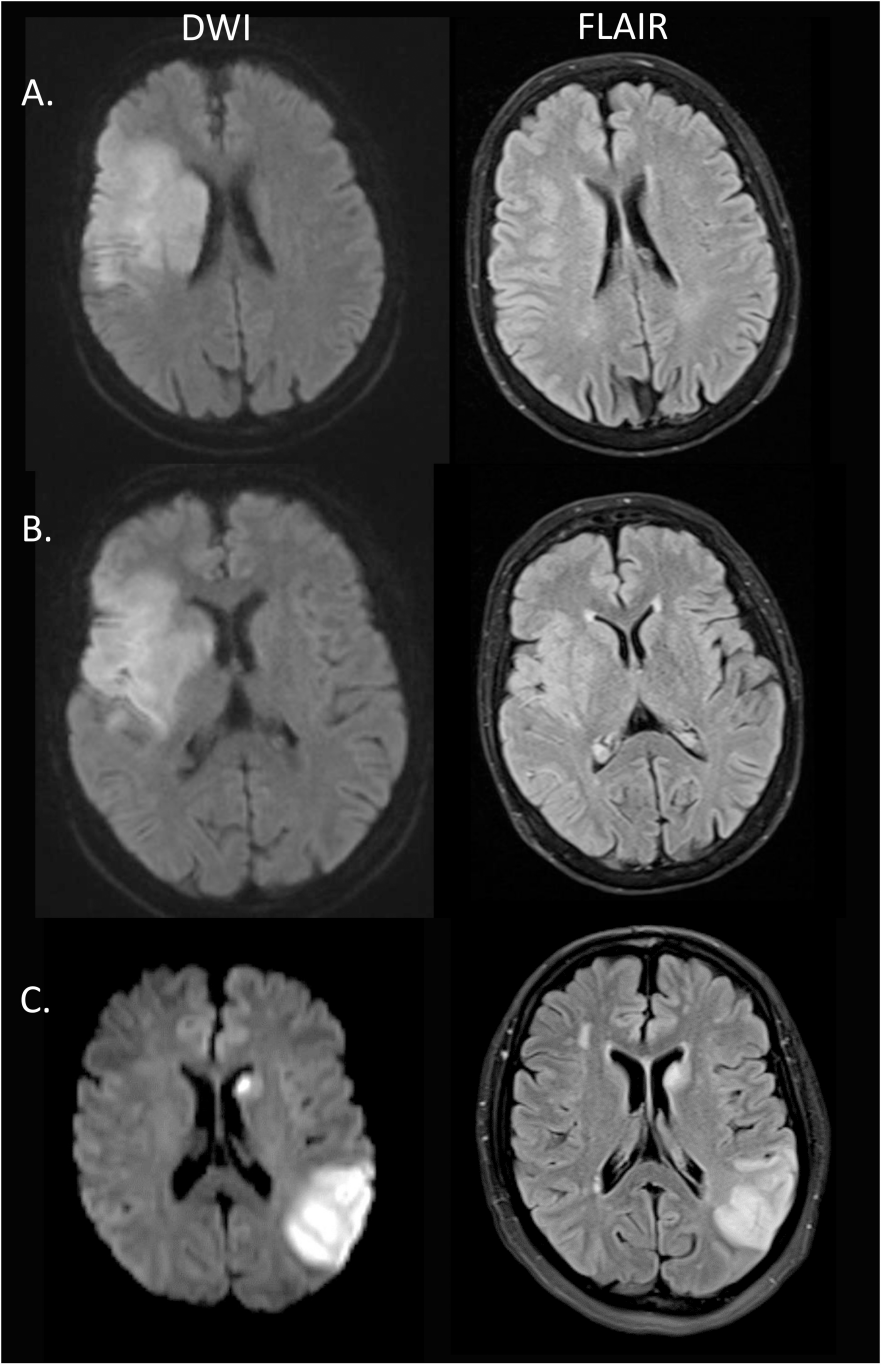
Exemplary illustration of variable amounts of FLAIR hyperintensity within the region of DWI lesion. A. Subtle focal FLAIR hyperintensity. B. Focal FLAIR hyperintensity of about 50% of the DWI lesion. C. Large (>50%) area of FLAIR hyperintensity within the DWI lesion. Patient C has been excluded from therapy, whereas patients A+B have been treated.

The treating neurologist and neuroradiologist decided whether to perform IVT, EVT or bridging both therapies on a case-to-case basis considering the patient’s age, past medical history, severity of stroke, and radiological findings. In particular, treatment was usually withheld if there was no relevant diffusion-perfusion mismatch on MRI or if the T2-weighted or FLAIR images revealed a hyperintense signal in more than 50% of the DWI lesion (as estimated by eyeballing)(Fig 1).

Age, gender, National Institutes of Health Stroke Scale (NIHSS) score, time from symptom onset to treatment, atrial fibrillation, hypertension, diabetes, smoking, hypercholesterolemia, treatment details (use of rt-PA, urokinase, mechanical procedures, bridging concept) and complications were recorded as baseline characteristics. Clinical outcome was assessed 3 months after the stroke using the modified Rankin scale (mRS). The study was performed with approval of the local ethics committee of Bern. The data was fully anonymized before scoring and analysis.

### MRI methods and image analysis

Pre-treatment MRI was performed using a 1.5T or 3T MR imaging system (Magnetom Avanto and Magnetom Verio; Siemens, Erlangen, Germany). The MRI protocol included whole brain DWI (parameters for 1.5T: TR 3000ms, TE 89ms, number of averages 4, FOV 230x230, voxel 1.2x1.2x5mm, slice thickness 5mm, matrix 192x192; for 3T: TR 3500ms, TE 89ms, number of averages 4, FOV 230x230, voxel 1.8x1.8x5mm, slice thickness 4mm, matrix 128x128) yielding isotropic b0 and b1000 as well as apparent diffusion coefficient (ADC) maps that were calculated automatically. We routinely included T2 weighted imaging (T2 parameters for 1.5T: TR 4000ms, TE 99ms, number of averages 1, FOV 230x230, slice thickness 5mm, Flip angle 143; T2 parameters for 3T: TR 4000ms, TE 118ms, number of averages 1, FOV 220x192, slice thickness 5mm, Flip angle 124) in our stroke imaging protocol until 2011 and replaced it with FLAIR imaging afterwards (FLAIR parameters for 1.5T: TR 8500ms, TE 88ms, TI 2440ms, number of averages 1, FOV 192x220, slice thickness 5mm, Flip angle 150; FLAIR parameters for 3T: TR 8500ms, TE 111ms, TI 2440ms, number of averages 1, FOV 192x220, slice thickness 5mm, Flip angle 150). Pre-treatment T2 weighted and FLAIR images were analysed for the presence of parenchymal infarct demarcation in the area of diffusion restriction on DWI by two raters (J.M. and P.P.K. for T2, J.M. and L.L. for FLAIR). Demarcation was defined on T2 weighted or FLAIR images as areas with signal increase compared to the contralateral non-affected anatomical structure. Raters were blinded to clinical outcome and bleeding complications. Anatomical localization of parenchymal infarct demarcation was graded according to the Alberta Stroke Program Early CT Score (ASPECTS).[23] Disagreements in scoring were resolved by discussion.

A CT or MRI control scan was obtained 24 to 72 hours after treatment or in any case of clinical deterioration. Symptomatic and asymptomatic intracerebral haemorrhage or hemorrhagic transformation (sICH/aICH) were graded according to the definition of the PROACT II Study.[24] The grading of bleedings was reviewed by an experienced neuroradiologist (L.L.). Hemorrhagic transformation was differentiated from retained contrast agent from the previous DSA by analysis of all available follow up images.

### Statistical analysis

Statistical analysis was performed using SPSS 21 (SPSS Inc., Chicago, Illinois, USA). Bivariable analysis of categorical variables was performed with χ2 and Fisher’s exact test as appropriate and continuous variables with Mann-Whitney test. Outcome was dichotomized into favorable (mRS 0–2) or poor clinical outcome (mRS 3–6). Forward stepwise logistic regression including all variables with p<0.2 in bivariate analysis (age, gender, time to thrombolysis, NIHSS score on admission, T2 or FLAIR hyperintensity, atrial fibrillation, diabetes, hypertension, hypercholesterolemia, smoking, occlusion type, for outcome also bleeding complications) was used to determine the predictors of bleeding complications and clinical outcome. The predictors of outcome were also determined by ordinal regression analysis in order to rule out false results due to lost power by dichotomization. All analyses were performed with group variables for T2 and FLAIR hyperintensities (any hyperintensity, basal ganglia involvement, cortex involvement), detailed with the anatomical regions according to the ASPECT scoring system and with the ASPECT score.

## Results

Baseline characteristics, treatment details and outcome of the 466 included patients are listed in Table 1. 307 patients received T2 weighted and 159 FLAIR imaging prior to treatment. 65 (14%) patients received IVT, 295 (63.3%) EVT and 106 (22.7%) bridging therapy (IVT followed by mechanical thrombectomy). Control imaging was CT in 68%, MRI in 19% and both CT and MRI in 14%. Symptomatic ICH occurred in 29 (6.2%) of the patients and any ICH in 95 (20.4%) patients. Outcome at 3 months was favorable in 225 (51.4%) patients.

**Table 1:** Baseline characteristics and outcome of 466 treated patients with anterior circulation stroke (n (%) of 466 patients if not stated otherwise).

Age, years (SD)	67.5 (14.7)
Women	218 (46.8)
**Vascular risk factors**	
- Hypertension	300/464 (64.7)
- Diabetes mellitus	74/465 (15.9)
- Atrial fibrillation	171/411 (41.6)
- Current smoking	104/426 (24.4)
- Hypercholesterolemia	257/460 (55.9)
Baseline NIHSS score, median (range)	12 (0–36)
**Vessel occlusion location**	
- Internal carotid artery	136 (29.2)
- Middle cerebral artery	308 (66.1)
- Anterior cerebral artery	4 (0.9)
- No visible vessel occlusion on angiogram	18 (3.9)
**Baseline imaging**	
- T2 weighted imaging	307 (65.9)
- FLAIR imaging	159 (34.1)
**Treatment type**	
- IVT	65 (14)
- EVT	295 (63.3)
- Bridging therapy	106 (22.7)
Minutes from symptom onset to treatment, median (range)	253 (45–1092)
**Outcome at 3 months**	
- mRS 0–2	225/438 (51.4)
- Survival	362/438 (82.6)
**Complications**	
- Any ICH	95 (20.4)
- Symptomatic ICH according to PROACT II	29 (6.2)

NIHSS: National Institutes of Health Stroke Scale; IVT: intravenous thrombolysis; EVT: endovascular treatment, mRS: modified Rankin Scale, ICH: intracerebral hemorrhage

The interrater agreement for rating hyperintensities on T2 weighted and FLAIR images was fair with a Cronbachs Alpha of 0.509 respectively 0.510.

The frequency of T2 or FLAIR hyperintensity within the acute DWI area and the association with ICH and outcome are listed in Table 2. Hyperintensities were found in 142 of 307 (46.3%) patients with T2 weighted imaging and in 89 of 159 (56%) patients with FLAIR weighted imaging (p = 0.051). Basal ganglia involvement was more frequently observed in FLAIR (33.3%) than in T2 (22.1%) weighted imaging (p = 0.010).

**Table 2:** T2 and FLAIR hyperintensity dependent bleeding complications and 3 months outcome of 466 patients.

	T2 +	T2 basalganglia involvement	T2 -	FLAIR +	FLAIR basal ganglia involvement	FLAIR -
**N (%)**	142/307 (46.3)	68/307(22.1)	165/307(53.7)	89/159(56)	53/159(33.3)	70/159(44)
**Any ICH**	33/142(23.2)	23/68(33.8)	30/165(18.2)	20/89(22.5)	15/53(28.3)	12/70(17.1)
**SymptomaticICH**	8/142(5.6)	4/68(5.9)	12/165(7.3)	6/89(6.7)	3/53(5.7)	3/70(4.3)
**mRS 0–2**	63/137(46)	25/65(38.5)	89/159(56)	35/78(44.9)	20/45(44.4)	38/64(59.4)

ICH: intracerebral hemorrhage

In multivariable regression analysis, hyperintensity of basal ganglia on T2 weighted imaging was the only predictor of any ICH (33.8% in patients with basal ganglia hyperintensity vs. 18.2% in those without; p = 0.003, OR 2.543, 95% CI 1.387–4.663). Detailed analysis of the anatomical regions according to the ASPECT classification revealed hyperintensity in the lentiform nucleus as predictor of any ICH in multivariable regression analysis (p = 0.001, OR 2.737, 95% CI 1.474–5.082). Any ICH occurred in 16.7% of the patients without and in 35.5% of the patients with hyperintensity in the lentiform nucleus. In patients with FLAIR images no independent predictor of any ICH was found but there was a trend towards significance for basal ganglia involvement (p = 0.069) and lentiform nucleus involvement (p = 0.052). In multivariable regression analysis T2 or FLAIR hyperintensity was not associated with sICH. The ASPECT score for T2 respectively FLAIR hyperintensity was not associated with ICH or outcome.

Hyperintensity on T2 weighted or FLAIR imaging within the ischemic lesion was not associated with worse 3 months outcome in multivariable regression analysis with dichotomized outcome or in ordinal regression analysis (p = 0.245 and 0.152 in multivariable regression analysis).

Asymptomatic ICH was associated with worse outcome in univariate analysis (p = 0.018) and ordinal regression analysis (p = 0.028, together with, NIHSS, age and time from symptom onset to treatment) but not in multivariable regression analysis with dichotomized outcome (dropout of the model with p = 0.061).

## Discussion

The main finding of this study is that focal hyperintensity on T2 weighted imaging within the DWI lesion prior to stroke treatment doubled the risk for bleeding when the basal ganglia, especially the lentiform nucleus, were involved. Nevertheless, neither hyperintensity on T2 nor on FLAIR imaging were associated with worse outcome. Accordingly, focal T2 or FLAIR hyperintensity within the DWI lesion should not lead to exclusion of patients from therapy per se, but patients and treating physicians should be aware of the elevated bleeding risk when thrombolytic agents are used in basal ganglia involvement.

Previous studies of infarct demarcation on T2 weighted or FLAIR imaging and the association with bleeding complications have provided varying results. Jha et al. found the FLAIR ratio to be associated with any ICH (including hemorrhagic transformation (HT)) and sICH in conservatively treated patients.[12] In patients treated with IVT, one study described an association with sICH[9], two others found an association only with any ICH (aICH and sICH)[10, 11] and one study did not find any association[13].

In our cohort there were focal hyperintensities in 46.3% of T2 weighted images and in 56% of FLAIR images. These rates are higher than reported in previous studies, which might be due to the average longer time from symptom onset to imaging in our patients, who received mainly EVT.

Bleeding risk in dependence on the localisation of hyperintensities was not reported in previous studies. T2 hyperintensity in the basal ganglia prior to treatment turned out as the only predictor for any ICH (including HT) in our analysis, whereby the lentiform nucleus was the most critical anatomical region. Any ICH occurred in 16.7% of the patients without and in 35.5% of the patients with hyperintensity in the lentiform nucleus (p = 0.001). In patients with FLAIR imaging there was only a trend for more ICHs in patients with hyperintensity of the lentiform nucleus (p = 0.052), which might be explained by the mean later appearance of hyperintensity on T2 weighted than on FLAIR imaging, indicating a longer time from symptom onset to imaging in patients with T2 hyperintensity, and/or by the smaller number of patients included with FLAIR imaging.

T2 or FLAIR hyperintensities and sICH were not associated in our study. However, there are inherent difficulties to differentiate symptomatic and asymptomatic ICH in some patients. It remains open, if an ICH classified as asymptomatic is without any effect on outcome when the patient remains clinically stable after recanalization. It may well be that such a patient would have improved without ICH. Similar to previous studies our patients with aICH had worse outcomes than patients without aICH in ordinal regression analysis.[25, 26] Accordingly, thrombolytic agents may be used in patients with focal T2 or FLAIR hyperintensity in basal ganglia infarcts, but patients and treating physicians should be aware of the elevated bleeding risk and potential worse outcome even when ICH remains so-called asymptomatic.

To date, the effect of T2 or FLAIR hyperintensity on outcome has been addressed in 4 studies. Two studies found FLAIR hyperintensities to be associated with an adverse clinical outcome after IVT[2, 14], but another study using IVT and an additional study using EVT did not.[15]^,^[16] In this study T2 hyperintensities were associated with any ICH, but there was no association with worse outcome in our patients, of whom the majority received EVT. To date most authors consider focal hyperintensities within the ischemic lesion as a tissue marker indicating the elapsed time since symptom onset and use this information to exclude such patients from reperfusion therapy. However, our results question whether focal hyperintensities are a suitable marker for treatment selection, at least for EVT.

The most important limitation of our study is its retrospective character. Second, we did not treat patients with very large areas of T2 or FLAIR hyperintensities which may have introduced a selection effect in our study. Therefore, our study does not permit any conclusion on patients with very large areas of T2 or FLAIR hyperintensity within the DWI lesion (>50% of the DWI lesion). In addition, the restriction of analysis to patients with baseline MRI, who account for about half of all treated stroke patients in our department, may also contribute to a selection bias. Third. the accuracy of the classification of HT and ICH on follow up images is influenced by the imaging technique. HT may be overestimated with MRI because of its high sensitivity for blood products, especially when susceptibility weighted imaging is used. When CT is used, contrast agent trapped in the infarcted area after EVT could be falsely classified as HT and lead to overestimation of HT. Fourth, the interrater agreement for rating focal hyperintensities on T2-weighted or FLAIR imaging was only fair. Therefore, patients with focal hyperintensities should be excluded from therapy only with caution if at all.

## Conclusions

In conclusion, our data indicate that patients with focal T2 or FLAIR hyperintensities within the ischemic lesion have a higher risk of bleeding when the infarct involves the basal ganglia. Nevertheless, our study of patients who received predominantly EVT did not show any association of T2 or FLAIR hyperintensities and worse outcome. In addition, there was only a fair interrater agreement for rating focal hyperintensities on T2-weighted and FLAIR imaging. Therefore, our results question whether patients with focal T2 or FLAIR hyperintensities should be excluded from reperfusion therapy, especially from EVT. On the contrary, future treatment trials may include also patients with large areas of FLAIR hyperintensity to evaluate the treatment effect and hemorrhage risk.

## Supporting information

S1 File Minimal data set. Minimal data set (SPPS).
(SAV)
